# Episodic Evolution and Adaptation of Chloroplast Genomes in Ancestral Grasses

**DOI:** 10.1371/journal.pone.0005297

**Published:** 2009-04-24

**Authors:** Bojian Zhong, Takahiro Yonezawa, Yang Zhong, Masami Hasegawa

**Affiliations:** School of Life Sciences, Fudan University, Shanghai, China; McGill University, Canada

## Abstract

**Background:**

It has been suggested that the chloroplast genomes of the grass family, Poaceae, have undergone an elevated evolutionary rate compared to most other angiosperms, yet the details of this phenomenon have remained obscure. To know how the rate change occurred during evolution, estimation of the time-scale with reliable calibrations is needed. The recent finding of 65 Ma grass phytoliths in Cretaceous dinosaur coprolites places the diversification of the grasses to the Cretaceous period, and provides a reliable calibration in studying the tempo and mode of grass chloroplast evolution.

**Methodology/Principal Findings:**

By using chloroplast genome data from angiosperms and by taking account of new paleontological evidence, we now show that episodic rate acceleration both in terms of non-synonymous and synonymous substitutions occurred in the common ancestral branch of the core Poaceae (a group formed by rice, wheat, maize, and their allies) accompanied by adaptive evolution in several chloroplast proteins, while the rate reverted to the slow rate typical of most monocot species in the terminal branches.

**Conclusions/Significance:**

Our finding of episodic rate acceleration in the ancestral grasses accompanied by adaptive molecular evolution has a profound bearing on the evolution of grasses, which form a highly successful group of plants. The widely used model for estimating divergence times was based on the assumption of correlated rates between ancestral and descendant lineages. However, the assumption is proved to be inadequate in approximating the episodic rate acceleration in the ancestral grasses, and the assumption of independent rates is more appropriate. This finding has implications for studies of molecular evolutionary rates and time-scale of evolution in other groups of organisms.

## Introduction

The grass family, Poaceae, is one of the largest plant families, comprising about 10,000 species including the most important agricultural plants, rice, wheat and maize, and grass-dominated ecosystems comprise about one-third of Earth's vegetative cover and support a vast number of animals [1]. It has long been suggested that the chloroplast (chl) genomes of the grass family have undergone an elevated evolutionary rate compared to other angiosperms [2]–[5], yet little is known when, why and how this rate change occurred.

To examine how the rate change occurred during evolution, it is prerequisite to know the time-scale of evolution. It has become increasingly feasible to estimate the phylogenetic tree and the time-scale of Angiosperm evolution by using chl genome sequences [5]–[9]. A reliable calibration is necessary to obtain reliable time estimates, but lack of good fossil evidence of the ancestral grasses has prevented us from addressing this issue. The recent finding of 65 Ma grass phytoliths in Cretaceous dinosaur coprolites [10], [11] places the diversification of the grasses to the Cretaceous period, and provides a reliable calibration in studying the tempo and mode of grass chl evolution. By using this calibration, we here find that episodic rate acceleration occurred in the common ancestral branch of the core Poaceae (a clade formed by rice *Oryza*, wheat *Triticum*, maize *Zea*, and their allies) accompanied by adaptive evolution in several chl proteins, while the rate reverted to the slow rate typical of most monocot species in the terminal branches. We also find that the widely used method for estimating divergence times based on the assumption of correlated rates between ancestral and descendant lineages [12]–[14] proved to be inadequate in approximating the process of grass chl evolution, and the assumption of independent rates [15] is more appropriate to studies of rate change over time. These results have implications for studies of molecular evolutionary rates and time-scale of evolution in other groups of organisms.

## Results

### Estimation of time-scale and pattern of rate change

**Figure 1:**
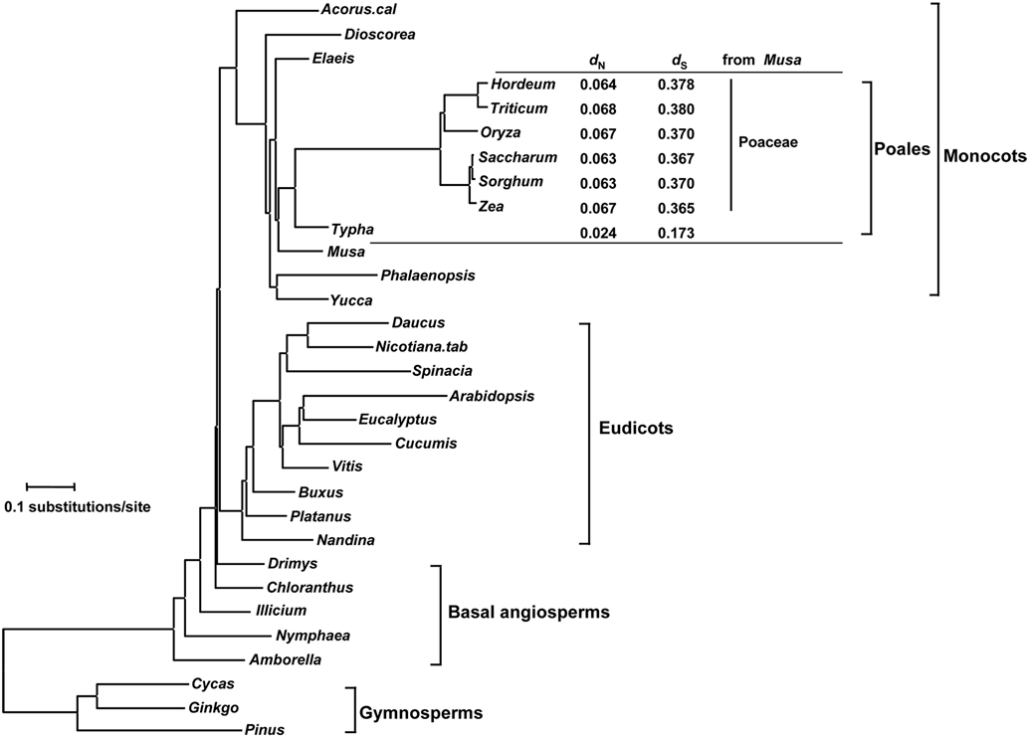
The phylogenetic tree of chloroplast genomes for the 31 species. The tree topology in Fig. 3 of ref.[6] was used, and the branch lengths were estimated by the ML with the codon-substitution model [21], [22] (CODEML in PAML [19]). The root was arbitrarily placed between Gymnosperm and Angiosperm. Non-synonymous (*d*_N_) and synonymous (*d*_S_) distances of Poales from *Musa* were estimated by CODEML.

Fig. 1 shows the ML phylogenetic tree of Angiosperm chl with Gymnosperm as an outgroup. The elongated branches of Poaceae are in accord with their widely accepted rate acceleration [2]–[5]. The global clock model in Poales (including Poaceae and *Typha*)+*Musa* was rejected when compared with the 2-local-clocks model (Poaceae lineages have a different rate from basal lineages such as *Typha* and *Musa*) by the likelihood ratio test (LRT) (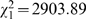, 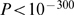 with the codon-substitution model). Moreover, longer distances of the Poaceae species from *Musa* than the *Typha/Musa* distance both in terms of non-synonymous and synonymous substitutions (Fig. 1) indicate that both types of substitutions have undergone rate acceleration along the line leading to Poaceae.

**Figure 2:**
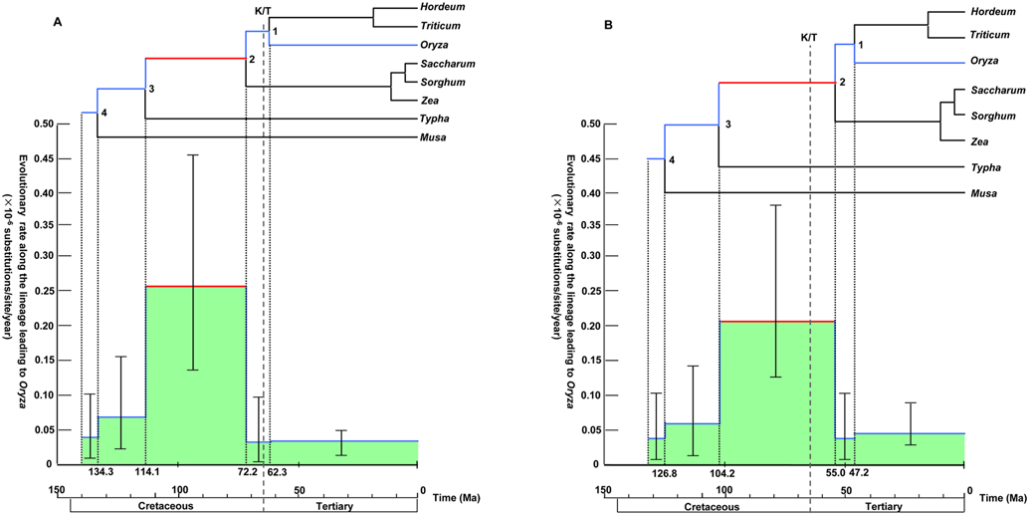
Posterior estimates of divergence times and rate change during evolution. Estimations were done by using MCMCTREE in PAML [19] with the IR model [15] for the rate change along lineages. Shape and scale parameters, α and β, in the gamma prior for parameter σ^2^ were 1.0 and 10.0, respectively. Only Poales+*Musa* part of the whole tree is shown, and a numbering of a node follows that of the whole tree in Fig. 3. The upper lines of the colored area trace the estimated rates along the lineage from the root to *Oryza* (the lineage indicated by colored lines in the tree) where 95% highest posterior density (HPD) is shown by a vertical line segment with two short horizontal line segments at boundaries. (A) >65 Ma constraint and (B) no constraint to the *Zea/Oryza* separation (for other calibrations, see Materials and Methods).

**Figure 3:**
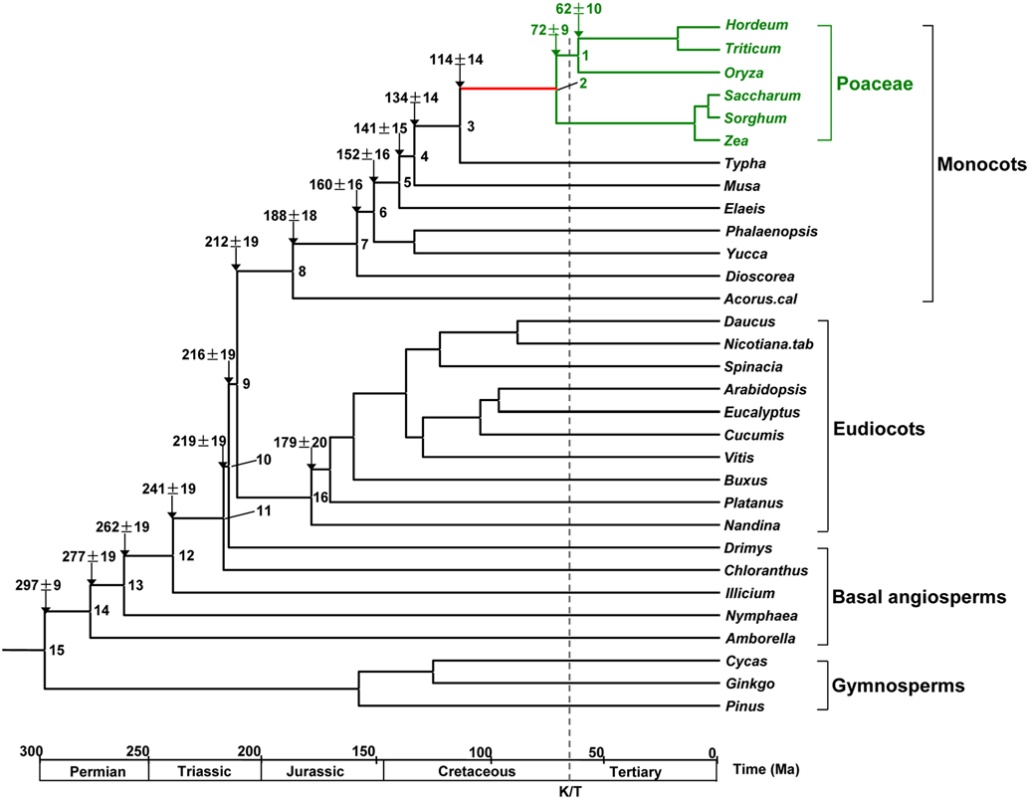
Posterior estimates of divergence times of a whole Angiosperm tree. Estimations were done by using MCMCTREE [19] with the IR model [15]. The >65 Ma constraint to the *Zea/Oryza* separation was applied.

To explore the pattern of rate change during the course of grass evolution in more detail, we estimated the time-scale of Angiosperm phylogeny, particularly focusing on monocots. Although several powerful methods have been developed for molecular time estimation allowing the rate change (a relaxed clock) [12]–[17], the poor quality of the fossil record for early grasses has prevented us from addressing this issue. Previously, the divergence among major groups of Poaceae was thought to have occurred in early Cenozoic, and the 60 Ma [18] and 50–60 Ma [5] date calibrations for the maize/wheat divergence were used in estimating the monocots/eudicots divergence time with chl DNA sequences. However, recent findings of grass phytoliths in Cretaceous dinosaur coprolites [10], [11] provided evidence that the major groups of core Poaceae had already diversified before Cretaceous/Tertiary (K/T) boundary of 65 Ma. Fig. 2A gives time estimates of the monocots evolution (Fig. 3 and Table 1 for the whole angiosperms) by a relaxed clock based on the Bayesian method implemented in MCMCTREE (in PAML [19]) with a constraint of >65 Ma for the *Zea/Oryza* divergence and with the independent-rates model for the rate change along lineages [15], [17]. In order to illustrate the rate change during evolution, a single instance of estimated rates along the lineage from the root to *Oryza* is also shown in Fig. 2A, where elevation of the rate only occurred on the common ancestral branch of Poaceae after they diverged from *Typha*.

**Table 1:** Posterior estimates of divergence times with the >65 Ma constraint to the *Zea/Oryza* separation.

Node	Independent-rates (IR) model		Correlated-rates (CR) model	
	Time (Ma)	Rate	Time (Ma)	Rate
1 (*Triticum*)	62.3 (44.4, 84.4)	0.033 (0.009, 0.090)	58.4 (45.5, 76.2)	0.030 (0.014, 0.058)
2 (*Zea*)	**72.2 (59.1, 93.7)**	**0.253 (0.137, 0.460)**	**67.7 (54.3, 86.9)**	**0.119 (0.087, 0.167)**
3 (*Typha*)	114.1 (91.4, 144.5)	0.069 (0.029, 0.157)	150.7 (123.2, 179.5)	0.062 (0.033, 0.104)
4 (*Musa*)	134.3 (111.9, 165.8)	0.040 (0.012, 0.106)	171.0 (145.1, 197.7)	0.048 (0.027, 0.079)
5 (*Elaeis*)	141.0 (117.0, 173.4)	0.046 (0.015, 0.116)	175.6 (149.3, 202.1)	0.065 (0.038, 0.104)
6 (*Phalaenopsis/Yucca*)	152.2 (125.7, 185.4)	0.041 (0.012, 0.109)	182.0 (156.0, 208.0)	0.047 (0.028, 0.076)
7 (*Dioscorea*)	159.6 (131.8, 193.6)	0.083 (0.035, 0.185)	187.0 (160.7, 212.9)	0.070 (0.043, 0.107)
8 (*Acorus*)	187.8 (153.1, 224.4)	0.057 (0.023, 0.136)	216.3 (188.8, 241.5)	0.053 (0.032, 0.085)
9 (Eudicots)	212.5 (175.0, 247.5)	0.035 (0.009, 0.096)	239.1 (211.5, 262.3)	0.055 (0.033, 0.086)
10(*Drimys*)	216.2 (178.0, 251.4)	0.033 (0.008, 0.091)	240.9 (213.4, 263.8)	0.050 (0.029, 0.079)
11(*Chloranthus*)	218.6 (180.1, 254.0)	0.061 (0.024, 0.145)	242.1 (214.6, 264.9)	0.055 (0.034, 0.086)
12(*Illicium*)	240.8 (199.7, 274.7)	0.053 (0.020, 0.125)	263.0 (235.3, 283.9)	0.056 (0.032, 0.091)
13(*Nymphaea*)	262.4 (219.8, 293.3)	0.043 (0.014, 0.108)	280.8 (253.3, 299.5)	0.057 (0.032, 0.092)
14(*Amborella*)	276.9 (233.3, 305.1)	0.054 (0.011, 0.174)	289.8 (262.2, 307.8)	0.059 (0.035, 0.096)
15(Gymnosperm)	297.2 (280.5, 310.3)	–	299.5 (281.4, 310.6)	–
16	179.4 (142.1, 218.8)	0.057 (0.024, 0.135)	196.7 (166.5, 230.0)	0.039 (0.025, 0.071)
Terminal branch to *Oryza*	0	0.038 (0.027, 0.052)	0	0.040 (0.030, 0.051)

MCMCTREE in PAML [19] was used with the GTR+Γ_5_ model. Shape and scale parameters, α and β, in the gamma prior for parameter σ^2^ were 1.0 and 10.0, respectively. 95% highest posterior density (HPD) is shown in parentheses. Rate (×10^−8^ substitutions/ nucleotide/year) refers to the rate of the branch preceding the node. Node numbers refer to those in Fig. 3, and taxa in parentheses refer to those branched off from the lineage leading to *Oryza*.

Although the fossil evidence for the >65 Ma constraint of the *Zea/Oryza* divergence is important in demonstrating the rate acceleration in ancestral grasses with subsequent slow-down, it is not a prerequisite. Even when the constraint was removed, almost the same pattern of rate change as that with the >65 Ma constraint was obtained when the IR model was used (Fig. 2B and Table 2), although the time estimate of the *Zea/Oryza* separation became younger (55.0 Ma). This time estimate is consistent with a conservative date of >50 Ma presented in refs. [5], [20], and our conclusion of the reverted slow rate in contemporary Poaceae can be regarded as robust to the calibration points used.

**Table 2 pone-0005297-t002:** Posterior estimates of divergence times without constraint to the *Zea/Oryza* separation.

Node	Independent-rates (IR) model		Correlated-rates (CR) model	
	Time (Ma)	Rate	Time (Ma)	Rate
1 (*Triticum*)	47.2 (28.4, 71.5)	0.040 (0.012, 0.105)	32.4 (21.6, 49.6)	0.059 (0.028, 0.103)
2 (*Zea*)	55.0 (35.2, 80.4)	0.212 (0.124, 0.377)	36.9 (24.6, 56.7)	0.119 (0.087, 0.159)
3 (*Typha*)	104.2 (78.7, 135.5)	0.063 (0.026, 0.144)	119.8 (90.9, 156.5)	0.059 (0.034, 0.096)
4 (*Musa*)	126.8 (105.7, 158.6)	0.040 (0.012, 0.105)	140.8 (113.0, 176.2)	0.043 (0.025, 0.069)
5 (*Elaeis*)	133.6 (111.1, 166.1)	0.044 (0.014, 0.112)	145.8 (117.4, 181.1)	0.057 (0.033, 0.091)
6 (*Phalaenopsis/Yucca*)	145.3 (120.2, 179.3)	0.041 (0.012, 0.108)	153.2 (124.5, 187.7)	0.043 (0.026, 0.066)
7 (*Dioscorea*)	152.6 (125.8, 187.8)	0.080 (0.034, 0.179)	158.7 (129.4, 193.2)	0.059 (0.035, 0.093)
8 (*Acorus*)	181.7 (147.1, 220.3)	0.054 (0.022, 0.128)	193.7 (159.4, 226.9)	0.046 (0.027, 0.073)
9 (Eudicots)	207.7 (169.2, 245.1)	0.036 (0.010, 0.098)	220.4 (184.1, 251.4)	0.047 (0.029, 0.072)
10 (*Drimys*)	211.3 (172.3, 249.1)	0.034 (0.009, 0.094)	222.6 (186.2, 253.2)	0.044 (0.026, 0.070)
11 (*Chloranthus*)	213.6 (174.3, 251.5)	0.060 (0.023, 0.141)	223.9 (187.4, 254.5)	0.047 (0.029, 0.072)
12 (*Illicium*)	236.2 (193.9, 272.9)	0.053 (0.020, 0.128)	248.6 (210.3, 277.3)	0.048 (0.027, 0.079)
13 (*Nymphaea*)	258.2 (213.6, 291.8)	0.042 (0.014, 0.107)	269.2 (229.4, 296.1)	0.048 (0.027, 0.078)
14 (*Amborella*)	272.8 (227.0, 304.2)	0.056 (0.012, 0.177)	279.7 (239.6, 305.8)	0.053 (0.031, 0.086)
15 (Gymnosperm)	296.6 (280.3, 310.2)	–	297.2 (280.4, 310.3)	–
16	176.9 (138.5, 217.4)	0.061 (0.026, 0.139)	178.5 (141.1, 215.1	0.040 (0.024, 0.068)
Terminal branch to *Oryza*	0	0.051 (0.032, 0.080)	0	0.074 (0.046, 0.106)

MCMCTREE in PAML [19] was used with the GTR+Γ_5_ model. Shape and scale parameters, α and β, in the gamma prior for parameter σ^2^ were 1.0 and 10.0, respectively. 95% HPD is shown in parentheses. Rate (×10^−8^ substitutions/ nucleotide/year) refers to the rate of the branch preceding the node. Node numbers refer to those in Fig. 3, and taxa in parentheses refer to those branched off from the lineage leading to *Oryza*.

In the first model of the relaxed clock implemented by Thorne and colleagues [12], [13], rates are auto-correlated between ancestral and descendant lineages on the tree, and the model is called the correlated-rates (CR) model. Sanderson's method of nonparametric rate-smoothing [14] was also based on the same idea. Later, an alternative model named the independent-rates (IR) model with no auto-correlation was developed [15], [17]. In Table 1, estimates of divergence times with the >65 Ma constraint for the *Zea/Oryza* separation are compared between the IR and CR models. The CR model tends to give older estimates for the nodes preceding the *Zea/Oryza* separation than the IR model. For example, the monocots/eudicots divergence time estimate was 239.1 Ma with the CR model, while the estimate with the IR model was 212.5 Ma which is more in accord with the recently published estimate of 140–150 Ma [5] even though it is still older. Without the >65 Ma constraint, the time estimate of the *Zea/Oryza* separation became too young (36.9 Ma) from the CR model to be compatible with the suggestion of >50 Ma from the previous works [5], [20], while the IR model gave compatible estimate of 55.0 Ma as mentioned before.

In order to examine the impact of including rapidly evolving Poaceae in the analysis, a comparison between the two models was carried out excluding Poaceae (Table 3). The time estimates were similar between the two models, and were similar to those from the IR model including Poaceae. For the monocots/eudicots separation, the IR model gave almost consistent estimates of 212.5, 207.7, and 216.5 Ma, respectively, with the >65 Ma constraint for the *Zea/Oryza* separation, without the constraint, and excluding Poaceae, while the CR model gave more diverged estimates of 239.1, 220.4, and 223.2 Ma, respectively. Interestingly, the estimates for this separation were very close between the two models when Poaceae species were excluded. This suggests that the episodic rate acceleration in ancestral Poaceae causes biased estimates, which the CR model cannot accommodate.

**Table 3:** Posterior estimates of divergence times excluding Poaceae.

Node	Independent-rates (IR) model		Correlated-rates (CR) model	
	Time (Ma)	Rate	Time (Ma)	Rate
4 (*Musa/Typha*)	115.5 (96.3, 136.7)	0.033 (0.011, 0.083)	110.6 (91.6, 128.6)	0.031 (0.016, 0.052)
5 (*Elaeis*)	125.1 (103.9, 149.2)	0.033 (0.012, 0.081)	119.2 (99.0, 138.7)	0.034 (0.022, 0.053)
6 (*Yucca/Phalaenopsis*)	139.5 (115.5, 167.3)	0.034 (0.010, 0.086)	130.8 (109.9, 150.3)	0.034 (0.021, 0.052)
7 (*Dioscorea*)	147.7 (122.0, 177.2)	0.060 (0.029, 0.128)	137.3 (115.4, 158.1)	0.037 (0.026, 0.056)
8 (*Acorus*)	184.6 (151.4, 219.8)	0.041 (0.019, 0.090)	190.8 (162.0, 218.1)	0.037 (0.022, 0.060)
9 (Eudicots)	216.5 (181.5, 248.9)	0.033 (0.010, 0.085)	223.2 (194.0, 246.9)	0.041 (0.027, 0.060)
10 (*Drimys*)	220.1 (184.7, 252.6)	0.031 (0.009, 0.081)	225.5 (196.2, 248.9)	0.041 (0.026, 0.062)
11 (*Chloranthus*)	222.2 (186.6, 254.9)	0.057 (0.024, 0.128)	226.7 (197.4, 250.2)	0.041 (0.028, 0.060)
12 (*Illicium*)	244.7 (206.9, 275.8)	0.050 (0.020, 0.114)	253.8 (222.5, 276.8)	0.045 (0.028, 0.070)
13 (*Nymphaea*)	266.3 (227.3, 294.5)	0.040 (0.014, 0.096)	274. 8 (241.9, 297.0)	0.044 (0.028, 0.068)
14 (*Amborella*)	280.2 (240.4, 305.9)	0.047 (0.011, 0.139)	285.2 (251.9, 307.0)	0.049 (0.031, 0.076)
15 (Gymnosperms)	298.3 (280.8, 310.4)	–	300.3 (282.0, 310.7)	–
16	190.4 (157.5, 223.6)	0.067 (0.031, 0.141)	188.6 (161.2, 213.2)	0.045 (0.030, 0.066)

MCMCTREE in PAML [19]] was used with the GTR+Γ_5_ model. Shape and scale parameters, α and β, in the gamma prior for parameter σ^2^ were 1.0 and 10.0, respectively. 95% HPD is shown in parentheses. Rate (×10^−8^ substitutions/ nucleotide/year) refers to the rate of the branch preceding the node. Node numbers refer to those in Fig. 3, and taxa in parentheses refer to those branched off from the lineage leading to *Oryza*.

In the above mentioned analyses, before fixing the shape and scale parameters (α and β) in the gamma prior for parameter σ^2^, which specifies how variable the rates are across branches, impact of priors on these parameters to posterior time and rate estimates was examined in detail (Tables S1, S2, S3, S4). Posterior time estimate for the *Zea/Oryza* separation depended less on the choice of the gamma prior for parameter σ^2^ with the IR model (Tables S1 and S3) than with the CR model (Tables S2 and S4), and therefore α and β in the gamma prior for parameter σ^2^ were arbitrarily chosen to be 1.0 and 10.0, respectively, in the analyses of Tables 1–3 and Figs. 2 and 3.

In order to further check the robustness of the time estimation on the choice of the substitution model, additional analyses based on a more realistic model of codon-substitution [21], [22] were carried out (Tables S5 and S6 with and without the >65 Ma constraint to the *Zea/Oryza* separation, and Table S7 excluding Poaceae). Comparisons of Tables S5 vs 1, Tables S6 vs 2, and Tables S7 vs 3 indicate that the estimated times are very similar between the two models, and that the estimated times are robust to the choice of a substitution model.

### Adaptive evolution

Non-synonymous/synonymous rate ratio (ω = d_N_/d_S_) is widely used as an indicator of adaptive evolution or positive selection [23]. Table 4 compares ω ratios along the branches estimated by different models. The minimum AIC [24] model shows that a pronounced increase of ω ratio occurred in the common ancestral lineage of Poaceae after they diverged from *Typha*, followed by reversion in the terminal branches to the lower level typical of basal lineages. The elevation of the ω ratio can be due either by adaptive evolution or by relaxation of selective constraints. A higher ω value than 1 is usually regarded as an evidence of adaptive evolution, but since the analysis shown in the table averages over the entire genomes, we would not get such a high value even if positive selection operated in some parts of some proteins. Therefore, the branch-site model [25], [26] was applied.

**Table 4:** Estimation of non-synonymous/synonymous rate ratio (ω) under different models by using CODEML in PAML [19].

Model	ω_0_	ω_1_	ω_2_	Ln L	LRT with 1ω-model	AIC
1ω	0.1518	–	–	−115,741.5	–	231,485.1
Simple 2ω	0.1265	0.1617	–	−115,729.5	9.63×10^−7^	231,463.0
3ω	0.1255	0.2189	0.1246	−115,669.4	4.95×10^−32^	231,344.8
Reverted 2ω	0.1250	0.2189	–	−115,669.4	3.29×10^−33^	***231,342.8***

1ω-model: (*Musa*#ω_0_, *Typha*#ω_0_, (crown Poaceae #ω_0_) #ω_0_).

Simple 2ω-model: (*Musa*#ω_0_, *Typha*#ω_0_, (crown Poaceae #ω_1_) #ω_1_).

3ω-model: (*Musa*#ω_0_, *Typha*#ω_0_, (crown Poaceae #ω_2_) #ω_1_).

Reverted 2ω-model: (*Musa*#ω_0_, *Typha*#ω_0_, (crown Poaceae #ω_0_) #ω_1_).

“Crown Poaceae” includes all Poaceae branches in our tree except for the common ancestral branch (stem Poaceae). The codon-substitution model with the F61 codon frequency was used. Minimum AIC (Reverted 2ω-model) is shown in bold italic.

To identify positively selected sites, among 61 protein-encoding “genes”, we at first selected 16 “genes”, for which the reverted 2ω-model (with the rate ratio ω_1_ of the common ancestral branch of Poaceae larger than the rate ratio ω_0_ of other branches) is significantly better than the 1ω-model (*P*<0.05) (Table 5), and by using the branch-site model [25], [26], we identified 5 genes (*atp*E, *cem*A, *clp*P, *rpo*B, and *rps*11) which have *P* value of the branch-site LRT less than 0.05 and contain positively selected sites (Table 6).

**Table 5:** LRT of 1ω-model vs reverted 2ω-model for individual genes.

Gene	Ancestral Poaceae 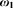	Other branches 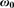	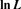 (reverted 2ω)	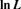 (1ω)	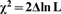	*P*
*rpo*B*	0.3469	0.1013	−8546.12	−8574.82	57.41	3.53E-14
*rps*11*	0.7406	0.0538	−1126.87	−1144.90	36.06	1.91E-09
*clp*P*	0.5943	0.1374	−1767.99	−1778.73	21.47	3.95E-06
*atp*E*	0.9642	0.1309	−1132.80	−1143.33	21.05	4.48E-06
*rps*3*	0.5767	0.1273	−1958.45	−1968.07	19.24	1.15E-05
*cem*A*	1.4337	0.3047	−2214.75	−2224.29	19.09	1.25E-05
*rpl*22*	0.6319	0.1139	−1067.47	−1075.53	16.11	5.97E-05
*rpo*C1	0.2385	0.1294	−5702.89	−5708.31	10.85	0.000989
*atp*A	0.2296	0.1102	−4201.36	−4206.20	9.68	0.001860
PS13#	0.0540	0.1663	−3542.69	−3547.23	9.08	0.002578
*rps*2	0.3623	0.1288	−1886.14	−1890.56	8.83	0.002960
*rpo*C2	0.3364	0.2179	−10553.23	−10557.46	8.45	0.003642
*psa*C#	0.0001	0.0780	−603.22	−607.35	8.26	0.004047
*rbc*L#	0.0344	0.0937	−3805.48	−3809.48	8.00	0.004669
*ndh*H	0.1405	0.0592	−3144.67	−3148.58	7.81	0.005202
*rps*7	1.2673	0.2151	−865.87	−869.76	7.78	0.005297
*rps*12	0.6567	0.1220	−697.24	−700.69	6.91	0.008589
*rps*19	0.6328	0.1836	−727.58	−730.62	6.08	0.013646
*rpl*16#	0.0268	0.1060	−1131.04	−1134.08	6.07	0.013739
*rpl*14	0.2858	0.0995	−972.76	−975.00	4.47	0.034457

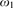
 and 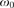 refer to the estimates of non-synonymous/synonymous rate ratios based on the reverted 2ω-model. Only 20 genes rejecting the 1ω-model with P<0.05 are listed. In this analysis, 11 monocot species (6 Poacea species, *Typha*, *Musa*, *Elaeis*, *Phalaenopsis*, and *Yucca*) were used. 75 genes were analyzed, but genes with related functions were concatenated if the lengths are shorter than 180 nucleotides, and therefore the number of tests was 61. PS13 refers to the concatenated sequences of *pet*G+*pet*L+*pet*N+*psa*I+*psa*J+*psb*F+*psb*I+*psb*J+*psb*K+*psb*L+*psb*M+*psb*N+*psb*Τ.

* refers to a gene which remains significant after the Bonferroni correction.

# refers to a gene with ω_1_<ω_0_.

**Table 6:** Branch-site test of positive selection.

gene	LRT	Positively selected sites
*atp*E	0.0484	2T→K, 17S→C, 41A→N, *64M→W*, 132V→W
*cem*A	0.0021	55N→R, 76Y→K, 161W→F, 190I→F, 204I→C
*clp*P	0.0081	26R→V, 48V→T, 86F→T, 112I→P, 134E→R, 182T→D
*rpo*B	0.0352	90R→F, 338G→K, 1026G→N
*rps*11	0.0082	*54V→P*, 62A→S, *82A→R*, *105L→S*, *115R→A*, 120L→R

The numberings of amino acids are those of *Zea mays* [45]. Positively selected sites were inferred at *P*_b_ = 95% with those reaching 99% shown in bold italic. The analyses were carried out for the 16 genes selected in Table 5, and only genes with positively selected sites and with *P*<0.05 (LRT) are listed.

Among the 16 genes with significantly higher ω_1_ than ω_0_ in Table 5 and among the 5 genes with positively selected sites in Table 6, only *atp*E is among the 14 genes with significant heterogeneity of nucleotide substitution rates for maize vs. rice, maize vs. wheat, or rice vs. wheat comparisons listed in Table 5 of ref. [27]. Four “genes”, *psa*C, *rbc*L, *rpl*6, and PS13, have significantly lower ω_1_ than ω_0_ (stronger purifying selection in the ancestral branch of Poaceae than in other branches). In Table 5, we carried out multiple tests for 61 “genes”. The Bonferroni correction is a safeguard against multiple tests falsely giving the appearance of significance, since 1 out of every 20 hypotheses tests is expected to be significant at the 5% level purely by chance. After performing the Bonferroni correction, 7 genes with * in Table 5 remained significant, that means, all the genes listed in Table 6 remained significant even by the conservative test of Bonferroni. On the other hand, the 4 “genes” with lower ω_1_ than ω_0_ in Table 5 were not significant after the Bonferroni correction.

## Discussion

In our study, the IR model gives more consistent results than the CR model, which has been widely used in estimating divergence times [9], [12]–[14], [28]–[34]. A basic assumption of the CR model is that rates change gradually over the tree. Our results suggest that the magnitude of the rate acceleration is underestimated by the CR model and that the IR model is more appropriate in approximating the rate change in the grass chl evolution. Although there exists a case in which the CR model outperforms the IR model [34], a number of authors have recently begun to notice that the IR model is superior to the CR model in approximating the evolution of evolutionary rates in several cases [17], [35]–[37].

Our analysis has revealed an episodic acceleration of the evolutionary rate of chl genomes during the emergence of core Poaceae, accompanied by adaptive evolution in several protein-encoding genes. Because the elevation of the rate occurred not only in non-synonymous substitutions but also in synonymous substitutions and because the elevated substitution rates were accompanied also by an elevated rate of insertions/deletions of nucleotides [9], the elevation of the mutation rate of chl genomes might have acted as a trigger of the adaptive evolution in the ancestral grasses, which might have facilitated the successful radiation and diversification of their descendants.

Suggested positive selection of *clp*P in *Oenothera* and *Sileneae* accompanied by elevated synonymous rate [38] might be related to our finding of rate acceleration in ancestral grasses both in terms of synonymous and non-synonymous substitutions. A more extensive study of chl genomes showed highly accelerated non-synonymous rates of ribosomal protein and RNA polymerase genes in Geraniaceae accompanied with the elevation of the ω ratio [39]. Interestingly, the 4 genes (*atp*E, *cem*A, *rpo*B, and *rps*11) detected to have positively-selected sites in our analysis (Table 6) are included in the gene group with significantly high ω ratio in Geraniaceae relative to other angiosperms (*clp*P was not analyzed in ref. [39]).

Recently, Smith and Donoghue [40] tested evolutionary rates across five groups of angiosperms, and found that the rates are generally low in trees/shrubs compared to related herbs. This is an interesting finding which links life history of plants to their rates of molecular evolution, and their conclusion generally holds in five different groups of Angiosperm. What we have shown in this work, however, is that the pattern of rate change during evolution is more complicated than has previously been anticipated. Our finding highlights the need for paying attention to rates of internal branches rather than averaging along a lineage in addressing the rate heterogeneity problem.

## Materials and Methods

Since our main interest was on grass evolution, we used all the monocot genera (13 species) and selected 18 species from outside monocots (31 species in total) among the 64 species in ref. [6]. We used 75 chl genes among 77 protein-encoding genes in ref. [6], excluding *inf*A and *ycf*2 because of missing data.

### Estimation of divergence times

The concatenated 75 gene sequences of chl from 31 species (from ref. [6]) and the tree topology in ref. [6] were used. To estimate divergence times and molecular evolutionary rates, a Bayesian method implemented in MCMCTREE (in PAML [19]) was applied either with the CR model [12], [13] or with the IR model [15] (using the GTR model with a discrete gamma distribution with five rate categories (Γ_5_) for nucleotide substitutions), and multiple calibrations were incorporated through the time prior. The Gymnosperm/Angiosperm divergence time was set at 280–310 Ma [5], [7]. Three nodes were constrained with minimum ages as follows; (1) the minimum age of the *Zea/Oryza* divergence was set either to 65 Ma [10], [11] or without this constraint, (2) >115 Ma constraint to the divergence of Poales from other monocots based on the earliest fossils of Poales [41], [42], and (3) >125 Ma for the most basal divergence in eudicots [7]. In order to check the robustness of the time estimation on the choice of the substitution model, the codon-substitution [21], [22]+Γ_5_ model was also used. The program adopts soft bounds, so that the probability that the true divergence time is outside the bounds is small but not zero [43]. In the Bayesian framework, priors are assigned not only on times, but also on the overall substitution rate parameter μ and on the rate-drift parameter σ^2^. So we roughly estimated the prior mean of the overall rate μ using the strict molecular clock with 295 Ma constraint to Gymnosperm/Angiosperm divergence time, and assigned the gamma prior G(4, 80) and G(4, 22) for this prior parameter in applying the nucleotide and codon substitution models, respectively. We next examined the impact of the rate-drift parameter σ^2^ by giving various priors for σ^2^ in applying the nucleotide substitution model. Posterior distributions of parameters were approximated using two independent MCMC analyses of 10^7^ steps each, following a discarded burn-in of 10^6^ steps. All the analyses were repeated with different inseed values to check for convergence of the MCMC chain.

### Non-synonymous/synonymous rate ratio

To the concatenated sequences of 75 protein-encoding genes of chl from 6 Poaceae species (*Oryza*, *Triticum*, *Hordeum*, *Zea*, *Saccharum*, and *Sorghum*), *Typha*, and *Musa*, we applied the codon-based likelihood models that allow for variable ω ratios among different lineages [44]. We used the likelihood ratio test (LRT) to compare the likelihood of one-ω ratio model, which assumes the same ω for all branch in the tree, with the two-ω ratio models, which assumes two different ω ratios. One of the two-ratio models (named “Simple 2ω-model”) assumes that Poaceae (including the common ancestral branch) has different ω from other parts of the tree as is represented by
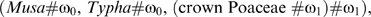
while the other (named “Reverted 2ω-model”) assumes that only the ancestral branch of Poceae has a different ω ratio than all the other branches in the tree as is represented by
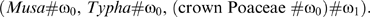
All the analyses were carried out with the CODEML program in PAML [19] using the codon-substitution model with the F61 codon frequency.

### Branch-site test of positive selection

The branch-site test was applied to the dataset of 11 monocot species in our dataset excluding the two most basal monocots; *i.e.*, *Dioscorea* and *Acorus*. The branch preceding the common ancestor of the core Poaceae was specified as a foreground branch, and all the others as background branches. LRT is constructed to compare an alternative model that allows for some codons under positive selection on the foreground branch with a null model that does not. The null model restricts codons on the foreground lineage to be undergoing neutral evolution (ω = 1). The specific codons which evolved under positive selection were identified on the foreground branch using a Bayes empirical Bayes procedure [25], [26].

## Supporting Information

Table S1 Impact of the shape and scale parameters (α and β) in the gamma prior for parameter σ^2^ using IR model with the >65 Ma constraint to the *Zea/Oryza* separation. 95% HPD is shown in parentheses. Times and rates are represented in 100 Ma (10^8^ years ago) and 10^−8^ substitutions/site/years, respectively.
(0.04 MB DOC)

Table S2 Impact of the shape and scale parameters (α and β) in the gamma prior for parameter σ^2^ using CR model with the >65 Ma constraint to the *Zea/Oryza* separation. 95% HPD is shown in parentheses. Times and rates are represented in 100 Ma (10^8^ years ago) and 10^−8^ substitutions/site/years, respectively.
(0.04 MB DOC)

Table S3 Impact of the shape and scale parameters (α and β) in the gamma prior for parameter σ^2^ using IR model without the constraint to the *Zea/Oryza* separation. 95% HPD is shown in parentheses. Times and rates are represented in 100 Ma (10^8^ years ago) and 10^−8^ substitutions/site/years, respectively.
(0.04 MB DOC)

Table S4 Impact of the shape and scale parameters (α and β) in the gamma prior for parameter σ^2^ using CR model without the constraint to the *Zea/Oryza* separation. 95% HPD is shown in parentheses. Times and rates are represented in 100 Ma (10^8^ years ago) and 10^−8^ substitutions/site/years, respectively.
(0.04 MB DOC)

Table S5 Posterior estimates of divergence times by MCMCTREE in PAML [19] using the codon-substitution+Γ_5_ model with the >65 Ma constraint to the *Zea/Oryza* separation. Shape and scale parameters, α and β, in the gamma prior for parameter σ^2^ were 1.0 and 10.0, respectively. 95% HPD is shown in parentheses. Rate (10^−8^ substitutions/codon/year) refers to the rate of the branch preceding the node. Node numbers refer to those in Fig. 3, and taxa in parentheses refer to those branched off from the lineage leading to *Oryza*.
(0.04 MB DOC)

Table S6 Posterior estimates of divergence times by MCMCTREE in PAML [19] using the codon-substitution+Γ_5_ model without constraint to the *Zea/Oryza* separation. Shape and scale parameters, α and β, in the gamma prior for parameter σ^2^ were 1.0 and 10.0, respectively. 95% HPD is shown in parentheses. Rate (10^−8^ substitutions/codon/year) refers to the rate of the branch preceding the node. Node numbers refer to those in Fig. 3, and taxa in parentheses refer to those branched off from the lineage leading to *Oryza*.
(0.04 MB DOC)

Table S7 Posterior estimates of divergence times by MCMCTREE in PAML [19] using the codon-substitution+Γ_5_ model excluding Poaceae. Shape and scale parameters, α and β, in the gamma prior for parameter σ^2^ were 1.0 and 10.0, respectively. 95% HPD is shown in parentheses. Rate (10^−8^ substitutions/codon/year) refers to the rate of the branch preceding the node. Node numbers refer to those in Fig. 3, and taxa in parentheses refer to those branched off from the lineage leading to *Oryza*.
(0.04 MB DOC)
