# Supplementary material for: Episodic Evolution and Adaptation of Chloroplast Genomes in Ancestral Grasses
Source: PLoS One. 2009 Apr 24;4(4):e5297. doi: 10.1371/journal.pone.0005297 (PMC2669172; doi:10.1371/journal.pone.0005297)
Supplement: Table S1 — Impact of the shape and scale parameters (α and β) in the gamma prior for parameter σ2 using IR model with the >65 Ma constraint to the Zea/Oryza separation. 95% HPD is shown in parentheses. Times and rates are represented in 100 Ma (108 years ago) and 10−8 substitutions/site/years, respectively. (0.04 MB DOC) [file pone.0005297.s001.doc]

**Table S1**.

| Prior  α | Prior  β | Posterior  σ^2^ | Posterior time: node38  (Monocot/  Eudicot) | Posterior time:  node44 | Posterior time:  node45  (*Zea/Oryza*) | Posterior rate:  Ancestral branch of node45 | Posterior rate:  *Oryza* |
| --- | --- | --- | --- | --- | --- | --- | --- |
| 0.1 | 0.1 | 0.4909  (0.3020, 0.7870) | 2.1344  (1.7644, 2.4974) | 1.1445 (0.8991, 1.4599) | 0.7273 (0.5931, 0.9524) | 0.2563 (0.1372, 0.4867) | 0.0376 (0.0266, 0.0527) |
| 0.1 | 1.0 | 0.4808 (0.2967, 0.7631) | 2.1512  (1.7627, 2.5158) | 1.1539 (0.9048, 1.4838) | 0.7349 (0.5897, 0.9885) | 0.2563 (0.1350, 0.4895) | 0.0373 (0.0256, 0.0527) |
| 0.1 | 10.0 | 0.3930 (0.2615, 0.5791) | 2.1205  (1.7490, 2.4717) | 1.1391 (0.9126, 1.4356) | 0.7192 (0.5886, 0.9406) | 0.2508 (0.1402, 0.4509) | 0.0374 (0.0267, 0.0510) |
| 1.0 | 0.1 | 0.5198 (0.3162, 0.8391) | 2.1364  (1.7535, 2.4991) | 1.1454 (0.9018, 1.4719) | 0.7346 (0.5948, 0.9779) | 0.2623 (0.1361, 0.5109) | 0.0375 (0.0260, 0.0539) |
| 1.0 | 1.0 | 0.5084 (0.3122, 0.8127) | 2.1529  (1.7628, 2.5217) | 1.1549 (0.9024, 1.4892) | 0.7389 (0.5914, 0.9984) | 0.2595 (0.1347, 0.5070) | 0.0373 (0.0254, 0.0532) |
| 1.0 | 10.0 | 0.4082 (0.2691, 0.6045) | 2.1248  (1.7498, 2.4749) | 1.1409 (0.9140, 1.4447) | 0.7215 (0.5905, 0.9368) | 0.2527 (0.1373, 0.4595) | 0.0375 (0.0268, 0.0515) |
| 10.0 | 0.1 | 1.0627 (0.5736, 1.8230) | 2.2010  (1.7632, 2.6109) | 1.1878 (0.8731, 1.6103) | 0.8079 (0.6096, 1.1770) | 0.3143 (0.1284, 0.7828) | 0.0372 (0.0223, 0.0628) |
| 10.0 | 1.0 | 0.9730 (0.5395, 1.6464) | 2.1633  (1.7387, 2.5677) | 1.1625 (0.8716, 1.5562) | 0.7852 (0.6110, 1.0940) | 0.3050 (0.1331, 0.6942) | 0.0374 (0.0237, 0.0614) |
| 10.0 | 10.0 | 0.6244 (0.3965, 0.9429) | 2.1671  (1.7669, 2.5455) | 1.1621 (0.8926, 1.5169) | 0.7571 (0.5982, 1.0393) | 0.2723 (0.1338, 0.5656) | 0.0371 (0.0245, 0.0555) |
