# Supplementary material for: Episodic Evolution and Adaptation of Chloroplast Genomes in Ancestral Grasses
Source: PLoS One. 2009 Apr 24;4(4):e5297. doi: 10.1371/journal.pone.0005297 (PMC2669172; doi:10.1371/journal.pone.0005297)
Supplement: Table S2 — Impact of the shape and scale parameters (α and β) in the gamma prior for parameter σ2 using CR model with the >65 Ma constraint to the Zea/Oryza separation. 95% HPD is shown in parentheses. Times and rates are represented in 100 Ma (108 years ago) and 10−8 substitutions/site/years, respectively. (0.04 MB DOC) [file pone.0005297.s002.doc]

**Table S2**.

| Prior  α | Prior  β | Posterior   σ^2^ | Posterior time: node38  (Monocot/  Eudicot) | Posterior time:  node44 | Posterior time: node45  (*Zea/Oryza*) | Posterior rate:  ancestral branch of node45 | Posterior rate:  *Oryza* |
| --- | --- | --- | --- | --- | --- | --- | --- |
| 0.1 | 0.1 | 1.0965 (0.6344, 1.7917) | 2.3670  (2.0308, 2.6433) | 1.5023 (1.1865, 1.8666) | 0.7202 (0.5722, 0.9896) | 0.1287 (0.0859, 0.1964) | 0.0377 (0.0260, 0.0491) |
| 0.1 | 1.0 | 1.0273 (0.6049, 1.6507) | 2.3955  (2.0735, 2.6632) | 1.5275 (1.2063, 1.8829) | 0.7239 (0.5659, 1.0193) | 0.1248 (0.0854, 0.1886) | 0.0375 (0.0253, 0.0493) |
| 0.1 | 10.0 | 0.6925 (0.4524, 1.0192) | 2.3944  (2.1174, 2.6292) | 1.5145 (1.2396, 1.8168) | 0.6752 (0.5406, 0.8630) | 0.1177 (0.0856, 0.1650) | 0.0399 (0.0299, 0.0507) |
| 1.0 | 0.1 | 1.1646 (0.6737, 1.8945) | 2.3632  (2.0197, 2.6489) | 1.5071 (1.1826, 1.8873) | 0.7352 (0.5777, 1.0472) | 0.1306 (0.0867, 0.2015) | 0.0370 (0.0245, 0.0488) |
| 1.0 | 1.0 | 1.0916 (0.6408, 1.7525) | 2.3953  (2.0590, 2.6770) | 1.5245 (1.1978, 1.9003) | 0.7307 (0.5705, 1.0396) | 0.1267 (0.0851, 0.1941) | 0.0373 (0.0247, 0.0492) |
| 1.0 | 10.0 | 0.7182 (0.4694, 1.0555) | 2.3912  (2.1147, 2.6229) | 1.5068 (1.2323, 1.7951) | 0.6769 (0.5431, 0.8693) | 0.1191 (0.0870, 0.1667) | 0.0398 (0.0298, 0.0506) |
| 10.0 | 0.1 | 2.2394 (1.2834, 3.5801) | 2.3878  (1.9186, 2.7350) | 1.5495 (1.1178, 2.0695) | 0.8368 (0.6017, 1.3415) | 0.1485 (0.0824, 0.2728) | 0.0335 (0.0190, 0.0485) |
| 10.0 | 1.0 | 1.9913 (1.1506, 3.1401) | 2.3648  (1.9364, 2.7043) | 1.5017 (1.1222, 1.9649) | 0.7810 (0.5988, 1.1555) | 0.1446 (0.0839, 0.2530) | 0.0353 (0.0223, 0.0488) |
| 10.0 | 10.0 | 1.0417 (0.6821, 1.5085) | 2.3878  (2.0663, 2.6613) | 1.5155 (1.1987, 1.8632) | 0.7231 (0.5678, 1.0025) | 0.1264 (0.0864, 0.1894) | 0.0376 (0.0257, 0.0492) |
