# Supplementary material for: Episodic Evolution and Adaptation of Chloroplast Genomes in Ancestral Grasses
Source: PLoS One. 2009 Apr 24;4(4):e5297. doi: 10.1371/journal.pone.0005297 (PMC2669172; doi:10.1371/journal.pone.0005297)
Supplement: Table S3 — Impact of the shape and scale parameters (α and β) in the gamma prior for parameter σ2 using IR model without the constraint to the Zea/Oryza separation. 95% HPD is shown in parentheses. Times and rates are represented in 100 Ma (108 years ago) and 10−8 substitutions/site/years, respectively. (0.04 MB DOC) [file pone.0005297.s003.doc]

**Table S3.**

| Prior  α | Prior  β | Posterior   σ^2^ | Posterior time: node38  (Monocot/  Eudicot) | Posterior time:  node44 | Posterior time:  node45  (*Zea/Oryza*) | Posterior rate:  ancestral branch of node45 | Posterior rate:  *Oryza* |
| --- | --- | --- | --- | --- | --- | --- | --- |
| 0.1 | 0.1 | 0.4672 (0.2879, 0.7451) | 2.0740  (1.7006, 2.4469) | 1.0430 (0.7709, 1.3583) | 0.5560 (0.3447, 0.8319) | 0.2170  (0.1233, 0.4038) | 0.0511 (0.0308, 0.0831) |
| 0.1 | 1.0 | 0.4585 (0.2838, 0.7308) | 2.0855  (1.6982, 2.4661) | 1.0459 (0.7721, 1.3735) | 0.5590 (0.3492, 0.8316) | 0.2174  (0.1214, 0.4044) | 0.0508 (0.0307, 0.0823) |
| 0.1 | 10.0 | 0.3770 (0.2491, 0.5585) | 2.0600  (1.6985, 2.4277) | 1.0423 (0.7889, 1.3432) | 0.5466 (0.3529, 0.7931) | 0.2101  (0.1234, 0.3722) | 0.0510 (0.0320, 0.0796) |
| 1.0 | 0.1 | 0.4987 (0.3030, 0.8056) | 2.0950  (1.7023, 2.4822) | 1.0498 (0.7691, 1.3854) | 0.5660 (0.3495, 0.8473) | 0.2203  (0.1211, 0.4219) | 0.0505 (0.0303, 0.0827) |
| 1.0 | 1.0 | 0.4807 (0.2966, 0.7668) | 2.0729  (1.6911, 2.4493) | 1.0438 (0.7689, 1.3630) | 0.5583 (0.3466, 0.8346) | 0.2187  (0.1227, 0.4131) | 0.0511 (0.0305, 0.0835) |
| 1.0 | 10.0 | 0.3933 (0.2592, 0.5841) | 2.0771  (1.6922, 2.4512) | 1.0421 (0.7868, 1.3554) | 0.5501 (0.3518, 0.8044) | 0.2123  (0.1235, 0.3767) | 0.0509 (0.0317, 0.0800) |
| 10.0 | 0.1 | 1.0115 (0.5412, 1.7490) | 2.1238  (1.6965, 2.5347) | 1.0716 (0.7360, 1.4819) | 0.6267 (0.3447, 1.0071) | 0.2599  (0.1153, 0.6196) | 0.0495 (0.0263, 0.0911) |
| 10.0 | 1.0 | 0.9441 (0.5225, 1.6008) | 2.1424  (1.7080, 2.5648) | 1.0756 (0.7297, 1.4918) | 0.6201 (0.3438, 0.9926) | 0.2533  (0.1144, 0.5999) | 0.0496 (0.0264, 0.0915) |
| 10.0 | 10.0 | 0.6021 (0.3798, 0.9176) | 2.0902  (1.6983, 2.4821) | 1.0517 (0.7571, 1.3928) | 0.5752 (0.3426, 0.8718) | 0.2272  (0.1199, 0.4511) | 0.0508 (0.0293, 0.0861) |
