# Supplementary material for: Episodic Evolution and Adaptation of Chloroplast Genomes in Ancestral Grasses
Source: PLoS One. 2009 Apr 24;4(4):e5297. doi: 10.1371/journal.pone.0005297 (PMC2669172; doi:10.1371/journal.pone.0005297)
Supplement: Table S4 — Impact of the shape and scale parameters (α and β) in the gamma prior for parameter σ2 using CR model without the constraint to the Zea/Oryza separation. 95% HPD is shown in parentheses. Times and rates are represented in 100 Ma (108 years ago) and 10−8 substitutions/site/years, respectively. (0.04 MB DOC) [file pone.0005297.s004.doc]

**Table S4.**

| Prior  α | Priorβ | Posterior   σ^2^ | Posterior time: node38  (Monocot/  Eudicot) | Posterior time:  node44 | Posterior time: node45  (*Zea/Oryza*) | Posterior rate:  ancestral branch of node45 | Posterior rate:  *Oryza* |
| --- | --- | --- | --- | --- | --- | --- | --- |
| 0.1 | 0.1 | 0.8084 (0.4551, 1.3675) | 2.1784  (1.7955, 2.5173) | 1.1936 (0.8975, 1.5849) | 0.3966 (0.2510, 0.6442) | 0.1244  (0.0885, 0.1722) | 0.0699 (0.0405, 0.1042) |
| 0.1 | 1.0 | 0.7727 (0.4377, 1.2944) | 2.2040  (1.8156, 2.5418) | 1.2122 (0.9023, 1.6206) | 0.3999 (0.2491, 0.6637) | 0.1221  (0.0865, 0.1693) | 0.0696 (0.0395, 0.1051) |
| 0.1 | 10.0 | 0.5381 (0.3458, 0.8087) | 2.1894  (1.8417, 2.4905) | 1.1781 (0.9088, 1.5215) | 0.3580 (0.2442, 0.5253) | 0.1200  (0.0884, 0.1583) | 0.0757 (0.0494, 0.1070) |
| 1.0 | 0.1 | 0.8955 (0.4908, 1.5571) | 2.2104  (1.8037, 2.5656) | 1.2317 (0.9025, 1.6813) | 0.4242 (0.2535, 0.7354) | 0.1234  (0.0853, 0.1749) | 0.0665 (0.0355, 0.1033) |
| 1.0 | 1.0 | 0.8294 (0.4683, 1.3929) | 2.1885  (1.8081, 2.5251) | 1.2026 (0.8983, 1.5956) | 0.4051 (0.2505, 0.6758) | 0.1245  (0.0882, 0.1747) | 0.0689 (0.0388, 0.1044) |
| 1.0 | 10.0 | 0.5643 (0.3600, 0.8520) | 2.2044  (1.8412, 2.5138) | 1.1977 (0.9093, 1.5648) | 0.3694 (0.2457, 0.5667) | 0.1190  (0.0871, 0.1589) | 0.0739 (0.0459, 0.1062) |
| 10.0 | 0.1 | 2.0570 (1.0919, 3.4384) | 2.2102  (1.7012, 2.6332) | 1.2894 (0.8885, 1.8007) | 0.5742 (0.2850, 1.0324) | 0.1446  (0.0871, 0.2473) | 0.0518 (0.0250, 0.0930) |
| 10.0 | 1.0 | 1.7495 (0.9574, 2.8712) | 2.2361  (1.7489, 2.6424) | 1.3029 (0.8968, 1.8418) | 0.5520 (0.2797, 1.0136) | 0.1364  (0.0839, 0.2224) | 0.0536 (0.0255, 0.0942) |
| 10.0 | 10.0 | 0.8798 (0.5546, 1.3202) | 2.1764  (1.7865, 2.5237) | 1.1986 (0.8966, 1.5732) | 0.4061 (0.2513, 0.6622) | 0.1252  (0.0890, 0.1744) | 0.0687 (0.0395, 0.1043) |
