# Supplementary material for: Episodic Evolution and Adaptation of Chloroplast Genomes in Ancestral Grasses
Source: PLoS One. 2009 Apr 24;4(4):e5297. doi: 10.1371/journal.pone.0005297 (PMC2669172; doi:10.1371/journal.pone.0005297)
Supplement: Table S7 — Posterior estimates of divergence times by MCMCTREE in PAML [19] using the codon-substitution+Γ5 model excluding Poaceae. Shape and scale parameters, α and β, in the gamma prior for parameter σ2 were 1.0 and 10.0, respectively. 95% HPD is shown in parentheses. Rate (10−8 substitutions/codon/year) refers to the rate of the branch preceding the node. Node numbers refer to those in Fig. 3, and taxa in parentheses refer to those branched off from the lineage leading to Oryza. (0.04 MB DOC) [file pone.0005297.s007.doc]

**Table S7.**

| Node | Independent-rates (IR) model | | Correlated-rates (CR) model | |
| --- | --- | --- | --- | --- |
|  | Time (Ma) | Rate | Time (Ma) | Rate |
| 4 (*Musa/Typha*) | 113.8  (94.4, 133.4) | 0.113  (0.037, 0.284) | 109.7  (90.0, 127.4) | 0.102  (0.053, 0.171) |
| 5 (*Elaeis*) | 123.5  (102.3, 146.7) | 0.111  (0.039, 0.272) | 118.8  (98.1, 138.0) | 0.113  (0.071, 0.175) |
| 6 (*Yucca/Phalaenopsis*) | 137.4  (113.6, 164.0) | 0.119  (0.038, 0.300) | 130.0  (108.8, 149.4) | 0.112  (0.069, 0.173) |
| 7 (*Dioscorea*) | 146.5  (121.1, 175.2) | 0.202  (0.095, 0.429) | 137.9  (115.5, 158.7) | 0.124  (0.085, 0.187) |
| 8 (*Acorus*) | 181.3  (148.9, 215.0) | 0.138  (0.064, 0.309) | 188.7  (160.5, 215.1) | 0.125  (0.075, 0.202) |
| 9 (Eudicots) | 212.1  (178.6, 243.4) | 0.115  (0.034, 0.293) | 219.7  (191.3, 243.2) | 0.137  (0.092, 0.203) |
| 10 (*Drimys*) | 216.1  (182.0, 247.7) | 0.110  (0.032, 0.282) | 222.3  (194.0, 245.7) | 0.139  (0.089, 0.210) |
| 11 (*Chloranthus*) | 219.7  (185.1, 251.6) | 0.191  (0.080, 0.429) | 224.5  (196.0, 247.9) | 0.139  (0.093, 0.205) |
| 12 (*Illicium*) | 241.3  (204.6, 272.4) | 0.174  (0.071, 0.391) | 250.2  (220.4, 272.8) | 0.154  (0.095, 0.242) |
| 13 (*Nymphaea*) | 263.1  (224.8, 291.6) | 0.150  (0.056, 0.356) | 271.6  (240.1, 293.0) | 0.154  (0.095, 0.237) |
| 14 (*Amborella*) | 281.0  (241.9, 306.0) | 0.158  (0.037, 0.474) | 286.4  (254.5, 307.1) | 0.170  (0.106, 0.265) |
| 15 (Gymnosperms) | 298.5  (281.0, 310.5) | $-$ | 300.4  (282.2, 310.7) | $-$ |
| 16 | 186.6  (154.7, 218.1) | 0.224  (0.102, 0.473) | 186.7  (160.0, 210.5) | 0.154  (0.103, 0.225) |
